# Beta-Tocotrienol Exhibits More Cytotoxic Effects than Gamma-Tocotrienol on Breast Cancer Cells by Promoting Apoptosis via a P53-Independent PI3-Kinase Dependent Pathway

**DOI:** 10.3390/biom10040577

**Published:** 2020-04-09

**Authors:** Maya Idriss, Mohammad Hassan Hodroj, Rajaa Fakhoury, Sandra Rizk

**Affiliations:** 1 Department of Natural Sciences, School of Arts and Sciences, Lebanese American University, Byblos 36, Lebanon; mti483@student.bau.edu.lb (M.I.); mohammadhassan.hodroj@lau.edu (M.H.H.); 2 Department of Biological Sciences, Faculty of Science, Beirut Arab University, Beirut 11-5020, Lebanon; rfakhoury@bau.edu.lb

**Keywords:** vitamin E, beta-tocotrienol, gamma-tocotrienol, apoptosis, breast cancer

## Abstract

Studies on tocotrienols have progressively revealed the benefits of these vitamin E isoforms on human health. Beta-tocotrienol (beta-T3) is known to be less available in nature compared to other vitamin E members, which may explain the restricted number of studies on beta-T3. In the present study, we aim to investigate the anti-proliferative effects and the pro-apoptotic mechanisms of beta-T3 on two human breast adenocarcinoma cell lines MDA-MB-231 and MCF7. To assess cell viability, both cell lines were incubated for 24 and 48 h, with different concentrations of beta-T3 and gamma-T3, the latter being a widely studied vitamin E isoform with potent anti-cancerous properties. Cell cycle progression and apoptosis induction upon treatment with various concentrations of the beta-T3 isoform were assessed. The effect of beta-T3 on the expression level of several apoptosis-related proteins p53, cytochrome C, cleaved-PARP-1, Bax, Bcl-2, and caspase-3, in addition to key cell survival proteins p-PI3K and p-GSK-3 α/β was determined using western blot analysis. Beta-tocotrienol exhibited a significantly more potent anti-proliferative effect than gamma-tocotrienol on both cell lines regardless of their hormonal receptor status. Beta-T3 induced a mild G1 arrest on both cell lines, and triggered a mitochondrial stress-mediated apoptotic response in MDA-MB-231 cells. Mechanistically, beta-T3′s anti-neoplastic activity involved the downregulation of phosphorylated PI3K and GSK-3 cell survival proteins. These findings suggest that vitamin E beta-T3 should be considered as a promising anti-cancer agent, more effective than gamma-T3 for treating human breast cancer and deserves to be further studied to investigate its effects in vitro and on other cancer types.

## 1. Introduction

Cancer remains a fatal disease threatening human lives all over the world. Females are potential victims of breast cancer (BC), which ranks globally the second in women’s death after lung cancer [1]. Over decades, the classification of BC has been changed progressively based on a list of criteria such as tumor grade, tumor size, histological types, lymph node status, hormone receptors, and gene expression, and helped in recognizing prognosis variability and predicting clinical outcomes [2,3,4]. Being heterogeneous by nature, breast cancer constitutes a difficult challenge for both the patients and the researchers working in the field of therapeutic treatment development. Recent studies are exploring natural compounds and extracts that can provide good therapeutic anti-tumor effects with less toxicity than commonly used synthetic drugs [1].

Vitamin E is a liposoluble micronutrient found in several food products and is considered as an important supplement for human health. In fact, only plants and cyanobacteria can synthetize various isoforms of this vitamin that englobes 8 hydrophobic members divided into 2 subgroups: tocopherols and tocotrienols that both comprise four isoforms, namely α, β, γ, and δ [5,6]. Among the same subgroup, isomers differ in methyl groups’ number and location on a chromane ring structure where β and γ isomer’s rings being dimethylated, while those of δ, and α are mono- and tri-methylated respectively. Moreover, tocopherols are characterized by having a saturated fatty acid tail in contrast to an unsaturated tail in tocotrienols [7,8]. 

In general, tocopherols are abundant in the majority of vegetable oils such as corn-, olive-, sunflower-, linseed-, and rapeseed-oils [9] whereas tocotrienols’ content is much higher in cereal grains (such as rice bran, oat, and barley), seeds (such as annatto, flaxseed, and grapefruit seed oils), and palm oil [10,11,12]. Other plant-derived oils may contain tocotrienols in trace or disproportional amounts as compared to that of tocopherols such as olive-, maize- and sunflower oils [12]. 

Several isoforms of the vitamin E were reported to be endowed with plenty of benefits for human health. However, studies showed that the vitamin E stereochemistry plays a crucial role in its biopotency; structurally, tocotrienols have one chiral center (C2), while tocopherols have three chiral carbon atoms [13]. Researchers verified that human bodies have the ability to retain the 2R stereoisomers with preferably the RRR stereoisomer, the only natural form of α-tocopherol and to eliminate the 2S-α-tocopherol [14]. In addition, there is a wide controversial case when studying the effect of α-tocopherol on human prostate cancer where, in SELECT studies reported by Klein et al. [15,16], the all-rac-alpha tocopheryl acetate (a mixture of 8 isomeric configurations) is found to increase the incidence of this type of cancer, while other scientists has reported a protective effect of α-tocopherol against it [17,18]. One possible reason could be referred to the difference in R and S stereoisomers bioactivity leading to variable effects of the same vitamin E member.

In the last decades, literature is focusing on the importance of tocotrienols especially with several data showing superiority of tocotrienols as antioxidants compared to tocopherols [19,20], in addition to their role as anti-inflammatory [21,22], hypoglycemic [23], immuno-stimulatory [1], cardio-[24,25], hepato-[26], neuro-[27] and nephro-protective agents [28]. Moreover, many anti-cancer effects of tocotrienols have been documented in vitro and in vivo involving proliferation, metastasis, apoptosis and angiogenesis pathways [29,30]. 

Since 1992, Nesaretnam and his colleagues reported that the tocotrienol-rich fraction (TRF) contained in palm oil, which included α-TP, α-T3, γ-T3, and δ-T3 isomers, in respective approximate proportions of 32%, 25%, 29%, and 14%, was responsible to reduce chemically- induced mammary tumors in rats fed with palm oil [1]. TRF was also proved to induce anti-proliferative effects against BC cancer; Mosselman et al. had previously reported that TRF was able to inhibit MDA-MB-231 and MCF7 BC cell lines growth, and specified that γ-, and δ- isomers were the most efficient in this inhibitory effect [31]. Additionally, TRF was responsible for inducing apoptosis, in several types of human cancer including RKO colon cancer cells, 1321N1, SW1783, LN18 glioma cells, and LNCaP, DU145, PC-3 prostate cancer cells [32,33,34].

Gamma-tocotrienol, a highly bio-active isomer, has recently attracted researchers’ attention as an anti-cancer agent that exhibits effective potency in different cancer types as prostate [35], colon [36], liver [37] and breast [38,39]. As anti-tumorigenic, gamma-T3 has been also reported to act through diverse mechanisms including NF-kB, TGF-β, and P38 pathways inhibition [40,41], caspases activation and apoptosis induction, cyclin D1 and cyclin-dependent kinases (CDK) 2, 4 and 6 levels reduction [26], and Ras/Raf/MEK/ERK and PI3K/AKT/GSK pathways down-regulation [42,43,44].

In contrast, beta-T3 is another isomer with a di-methylated ring which has been rarely studied for its anti-cancer effects due to its low abundance in nature and the difficulty in its extraction as compared to other vitamin E derivatives. In a recent study, Lim et al. found that beta-T3, purified from palm oil, was able to inhibit the growth of a human lung cancer cell line A549 and a glioblastoma cell line U87MG and to induce apoptotic morphologies such as membrane blebbing, and chromatin condensation. Beta-T3 activity was proved to involve both the extrinsic and the intrinsic apoptotic pathways [45].

The current study aims to investigate the anti-proliferative effect of beta-tocotrienol on two human breast adenocarcinoma cell lines, MDA-MB-231 and MCF7, in comparison to the widely studied gamma-tocotrienol isoform. The mechanism of action of beta-T3 was further investigated using together flow cytometry, followed by western blot analyses to elucidate the underlying molecular pathways.

## 2. Materials and Methods 

### 2.1. Drugs

Beta-Tocotrienol (ab145176) and Gamma-Tocotrienol (ab144448) [C28H42O2; MW: 410.64] (2 R configuration for both compounds) were purchased from Abcam (Cambridge, UK) (purity >98%). 5 mg of beta T3 were dissolved in 1.22 mL of dimethyl sulfoxide (DMSO) to obtain 10,000 μM concentrated stock solution. The stock was stored at −20 °C until use. On the day of treatment, DMEM (Dulbecco’s Modified Eagle’s Medium with 4.5 g/L Glucose with L-Glutamine, Lonza) media was used to prepare different fresh dilutions to prepare 10, 20, 30, 40, and 50 μM (beta-T3) doses. While for gamma-T3, a fresh mixture with DMEM media was directly prepared on the day of treatment from the 10 mM DMSO stock of gamma-T3 followed by a similar range of concentrations used for treating BC cells (10 to 50 μM).

### 2.2. Cell Lines and Cell Culture Conditions

A triple-negative human BC cell line, (TNBC) MDA-MB-231 and an estrogen-positive BC (ER-+ve) cell line, MCF7 were purchased from the American Type Culture Collection (ATCC, Manassas, VA, USA) and were cultured in DMEM medium supplemented with 10% of fetal bovine serum FBS (Sigma Aldrich, St Louis, MO, USA), and 1% of 100 U/mL penicillin and 100 µg/mL streptomycin (Lonza, Allendale, NJ). Cells were grown at 37 °C in a humidified, 5% CO2 incubator.

### 2.3. In Vitro Cell Proliferation Assay

To assess cell proliferation, WST1 assay was performed in conformity with the manufacturer’s guide instructions (Roche, Penzberg, Germany) and as previously reported [46,47,48]. The assay is based on using the WST-1 tetrazolium salt that is cleaved to generate formazan dye once mixed with metabolically active cells. Cell number is then quantified by measuring formazan absorbance values at 450 nm by an ELISA reader. 

Using a seeding density of 2 × 10^5^ cells/mL and 10^5^ cells/mL, for MCF7 and MDA-MB-231 respectively, cells were plated in triplicates in 96-well plates and incubated for 24 h prior to drug treatment. The next day, a range of concentrations of beta-T3 or gamma-T3 was freshly prepared to treat the cells for 24 or 48 h; control cells treated with the vehicle (DMEM). After incubation for the desired treatment period, 10 μL of WST-1 were added to the cells in each well, and then absorbance values A_450_ were measured after two hours using a Varioskan Flash microplate ELISA reader (Thermo Fisher Scientific, Waltham, MA, USA). Proliferation percentages were calculated by dividing the average of the absorbance obtained for cells treated with beta-T3 by the average of the absorbance obtained for control cells treated with the vehicle. The reported data includes the results obtained from three independent experiments.

### 2.4. Cell Cycle Analysis

To detect the effect of beta-T3 on cell cycle progression, the cellular DNA content was analyzed using flow cytometry after being stained with propidium iodide, as previously described [49]. Cells were seeded in 6-well cell culture plates at a density of 10^5^ cells/mL for MDA-MB-231 and 2 × 10^5^ cells/mL for MCF7 for 24 h prior to drug treatment. After 24 or 48 h of exposure to beta-T3, cells were detached, collected in tubes, centrifuged for 5 min at 4 °C at 1500 rpm. Pellets were then re-suspended in 600 μL of cold phosphate-buffered saline (PBS) (BE17-516F, Lonza, Verviers Belgium), then fixed in 1400 μL of ice cold ethanol (97%) and then stored in a −80 °C freezer for at least 24 h prior to staining and analysis.

The tubes were then centrifuged for 5 min at 4 °C at 1500 rpm. 2 × 700 μL from the supernatant were discarded to be replaced by 500 μL of PI staining solution (1 × PBS, 1 mg/mL propidium iodide (Sigma Aldrich, St Louis, MO, USA) and 100 µg/mL RNase (Roche, Penzberg, Germany) in a volume proportion of 600:30:2 respectively, and then incubated for 40 min in dark before being analyzed by flow cytometry (BD Accuri C6; Becton-Dickinson, Biosciences, NJ, USA).

BD Accuri C6 Plus software was used to detect the cell DNA content. Based on DNA content, cells were distributed into their cell cycle phases: cells with < 2n were grouped into the sub-G0/G1 phase (G = Gap phase), those with 2n into the G0/G1 phase, those with a DNA content between 2n and 4n into the synthesis phase(S), and those with 4n into the G2/M phase. Cell death can be noticed by the increase in the pre-G phase in treated samples in comparison to the untreated control.

### 2.5. Cell Apoptosis Detection by Annexin V/PI Staining

MDA-MB-231 (S.D = 10^5^ cells/mL) and MCF7 (S.D = 2 × 10^5^ cells/mL) were cultured in 6-well cell culture plates. After incubation for 24 h at 37 °C in a humidified atmosphere with 5% CO2, treatment with beta-T3 (10, 20, 30, 40 and 50 μM) was performed, for both 24 and 48 h. Cells were washed, detached and centrifuged at 1500 rpm at 4 °C for 5 min. The pellet was resuspended in incubation buffer and stained with Annexin V and propidium iodide (PI) following the manufacturer’s instruction (Annexin V-FITC Apoptosis Detection Kit; Abcam). Cells were then analyzed by flow cytometer using the BD Accuri C6 Plus Software as previously described [50,51]. Living cells are stained negative for both Annexin-V and PI. Cells stained positive only for Annexin-V are early apoptotic, whereas those stained positive for both Annexin-V and PI are in the late apoptotic stage.

### 2.6. Protein Extraction and Western Blots

Western blot analysis was performed to assess the expression level of various targeted proteins: Bax, Bcl2, p53, cleaved PARP-1, cytochrome c, caspase-3, p-PI3K, and p-GSK-3α/β. Equal-loaded expression of beta-actin levels was certified to be taken as a reference for proteins level comparison. 

MDA-MB-231 cells were seeded into 6 well plates at a density of 10^5^ cells/mL. Cells, except the controls, were treated with 10, 20, and 30 μM of beta-tocotrienol after a 24 h incubation period (37 °C in a humidified atmosphere containing 5% CO2), After the desired period of treatment (24 h), the cellular proteins were extracted using Q-proteome Mammalian Protein kit (QIAGEN) as previously described [52]. Proteins were quantified using Lowry assay.

10% SDS-PAGE gels were prepared to separate protein samples using 120 V running voltage, and then transferred to PVDF activated membranes at 250 mA for 90 min. Blocking was performed with a 5% milk solution(skimmed milk+PBS+Tween-20), for 90 min at room temperature with a gentle shaking, or during overnight at 4 °C. Then, blocked membranes were incubated with primary antibodies prepared in 2% milk solution: Anti-Actin (Santa Cruz Biotechnology, sc-47778), anti-PARP-1 (ab32064), anti-Bax (abcam, ab182733), anti-p53 (Elabscience, E-AB-22015), anti-Bcl2 (ab692), anti- cleaved caspase-3 (ab13585), and anti-cytochrome C (Elabscience, E-AB-63402) for 1 h at room temperature with a gentle shaking. As well, primary anti-phosphorylated antibodies: anti-PI3K (ab182651) (phosphorylated on Tyrosine: Y607) and anti-GSK-3 α & β (ab68476) (phosphorylated on tyrosine Y 279 & tyrosine Y 216 respectively), were used in the same conditions except the replacement of skimmed milk by bovine serum Albumin (BSA) to reduce background and prevent milk casein reaction with these antibodies.

Membranes were then washed 3 times using a washing solution (1 × PBS and 0.5% Tween-20) before being incubated with appropriate HRP-conjugated secondary antibodies (anti-mouse antibody (W4028) or anti-rabbit antibody (W4018), in accordance to each used primary antibody for 1h, with a gentle shaking. The latter antibodies were provided by Promega (Madison, WI, USA). Then a second set of washings was done, to prepare membranes for imaging by the ChemiDoc XRS machine (Bio- Rad, Hercules, CA, USA). Addition of ECL (GE Healthcare, Buckinghamshire, UK) chemi-luminescent reagent was necessary to detect the bands. Image J software (version 1.52u, National Institute of Health, Wisconsin, Bethesda, Maryland, USA) was used for proteins quantification.

### 2.7. Statistical Analysis

All the reported results were expressed as mean value +/− standard deviation, between three different trials done for each experiment. One-way ANOVA test was used for analyzing differences between treatments, and Student’s T-test was performed to compare differences between treated and untreated control groups. The level of significance was set at *p* < 0.05 in comparing control values versus treated ones.

## 3. Results

### 3.1. Effect of Beta- and Gamma- Tocotrienols on the Cell Proliferation of MDA-MB-231 and MCF7 cells

**Figure 1:**
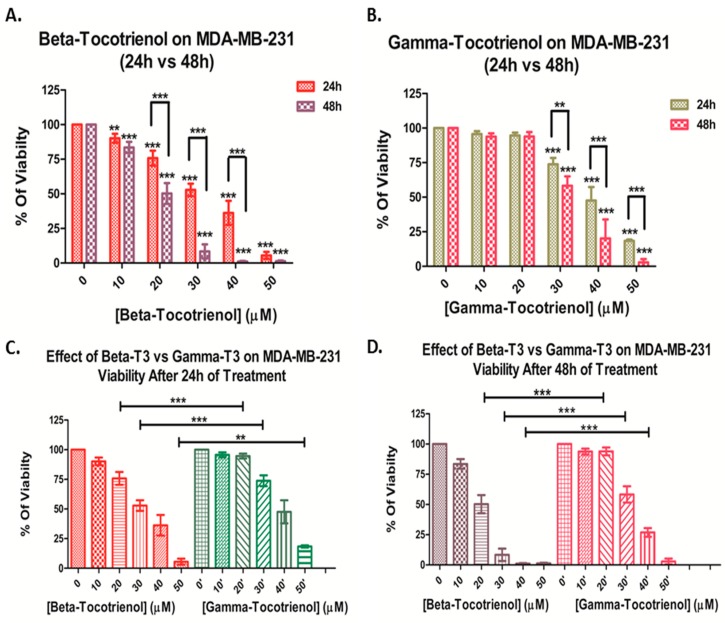
Proliferation of MDA-MB-231 cells after 24 and 48 h of treatment with various concentrations of beta- (**A**) and gamma-(**B**) tocotrienols (0–50 μM). Significance between both treatments was tested after 24 h (**C**) and 48 h (**D**). ** and *** indicate *p* <  0.001 and *p* <  0.0001 respectively.

Using WST-1 as a cell proliferation reagent, the percent proliferation of the MDA-MB-231 cell line treated with different concentrations of beta-T3 (10–50 µM) or gamma-T3 (10–50 µM) for 24 and 48 h was calculated and the results showed a significant dose- and time-dependent decrease in the proliferation of both cell lines; however, the effect was more prominent with beta-T3 treatment. Beta-tocotrienol induced a significant progressive decrease in percentage of proliferating MDA cells, with an IC50 of 29.99 μM and 21.14 μM after 24 and 48 h respectively (Figure 1A). On the other hand, gamma-tocotrienol induced a significant progressive decrease in cell proliferation of MDA cells starting from 30 μM with an IC50 of 39.04 μM and 30.98 μM after 24 and 48 h respectively (Figure 1B). The IC50 concentrations of beta-T3 were lower than that of the gamma derivative after both 24 and 48 h treatments, indicating a significant higher potency of beta-T3 on MDA cells at 20, 30 and 40 μM (Figure 1C,D).

**Figure 2:**
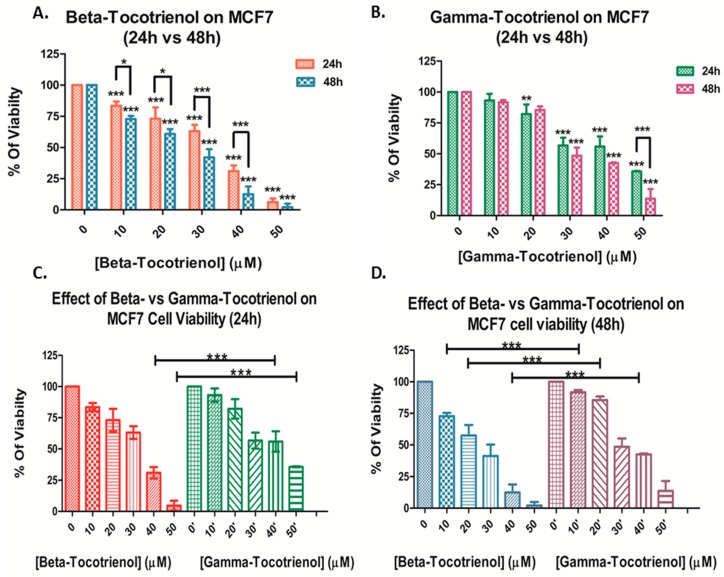
Proliferation of MCF-7 cells after 24 and 48 h of treatment with various concentrations of beta-(**A**) and gamma-(**B**) tocotrienols (0–50 μM). Significance between both treatments was tested after 24 h (**C**) and 48 h (**D**). *, ** and *** indicate *p* < 0.05, *p* < 0.001 and *p* < 0.0001 respectively.

Similarly, beta-T3 exhibited a significant dose- and time-dependent anti-proliferative effect on MCF cells, with an IC50 of 30.48 μM and 24.34 μM after 24 and 48 h respectively (Figure 2A). On the contrary, gamma-T3 induced a significant progressive decrease in cell proliferation of MCF cells starting from 20 μM with an IC50 of 41.05 μM and 32.87 μM after 24 and 48 h respectively (Figure 2 B). When compared to that of gamma-T3, the IC50 concentrations of beta-T3 were lower after both 24 and 48 h treatments, indicating a significant higher potency of beta-T3 on MCF cells upon treatment with 20, 30 and 40 μM of beta-T3 (Figure 2C,D).

Overall, upon comparison of the responses of both BC cell lines, the triple-negative BC cell line MDA-MB-231 was found to be more sensitive than the ER-positive MCF7 cell line, in response to both vitamin E tocotrienols, remarkably to beta-T3 that showed a similar pattern in both cell lines (Table 1). 

### 3.2. Effect of Beta-Tocotrienol on the Cell Cycle Progression of BC Cell Lines

To investigate whether the anti-proliferative effect of beta-T3 on both BC cells is due to a cell cycle arrest induction, propidium iodide staining was performed followed by flow cytometry analysis. 

**Figure 3:**
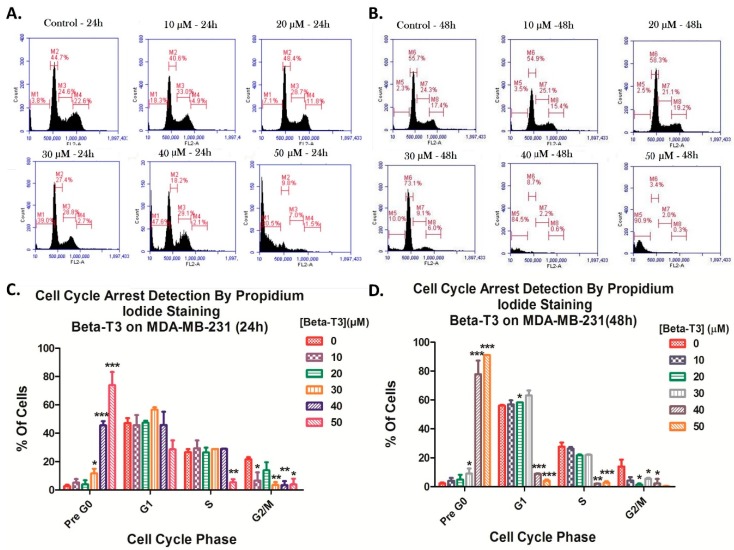
Beta-tocotrienol induced a mild cell cycle arrest of TNBC MDA-MB-231 cells. Cell cycle distribution was assessed by flow cytometry after propidium iodide staining using the range of concentrations: 0–50 μM for 24 (**A**) or 48 h (**B**). Figures (**A**) and (**B**) were obtained by the C Flow software (version 1.0.227.4, Accuri Cytometers, Ann Arbor, MI, USA). (**C**) and (**D**) represent histograms for analysis of the percentages of cells in each cell cycle phase upon treatment with beta-T3 for 24 and 48 h respectively. *, ** and *** indicate *p* < 0.05, *p* < 0.001 and *p* < 0.0001 respectively.

Comparing the MDA cells treated with beta-T3 for 24 h with the non-treated control cells showed a significant dose-dependent increase in the sub-G1 population from 3.8% in the control cells to 80.5% in cells treated with 50 μM, which may reflect an increase in cellular fragmentation. Whereas, after 48 h of treatment, the effect was significantly higher showing a time-dependent increase in sub-G1 population from 2.3% in control cells to 90.9% in cells treated with 50 μM. Moreover, beta-T3 treatment was found to induce a mild G1 phase arrest at the 20 μM dose after 48 h of treatment (Figure 3).

**Figure 4:**
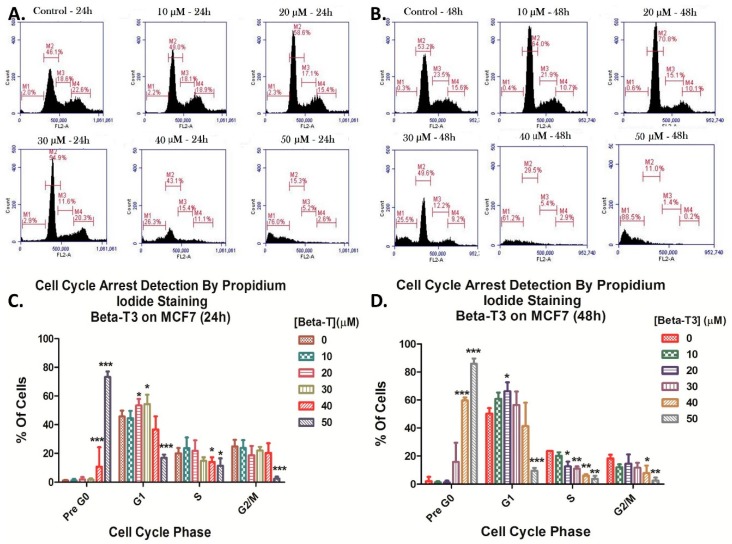
Beta-tocotrienol induced a cell cycle arrest of ER-(+ve) MCF7 cells. Cell cycle distribution was assessed by flow cytometry after propidium iodide staining using the range of concentrations: 0–50 μM for 24 (**A**) or 48 h (**B**). Figures (**A**) and (**B**) were obtained by the C Flow software. (**C**) and (**D**) represent histograms for analysis of the percentages of cells in each cell cycle phase upon treatment with beta-T3 for 24 and 48 h respectively. *, ** and *** indicate *p* < 0.05, *p* < 0.001 and *p* < 0.0001 respectively.

In comparison to MDA cells, MCF cells showed a significant cell accumulation in the G1 phase at both 20 and 30 μM doses (*p* < 0.05) upon 24 h treatment (Figure 4A,C) and only at the 20 μM dose (*p* < 0.05) upon 48 h treatment (Figure 4B,D). Similarly to its effect in MDAs, beta-T3 was found to induce cellular fragmentation of MCF7 cells with a significant dose- and time-dependent sub-G1 increase (*p* < 0.0001) from 2.0% to 76.0% and from 0.3%, to 88.5% between the control and 50 μM treated groups after 24 and 48 h of treatment respectively (Figure 4).

### 3.3. Beta-Tocotrienol Induces Apoptosis in BC Cell Lines

**Figure 5:**
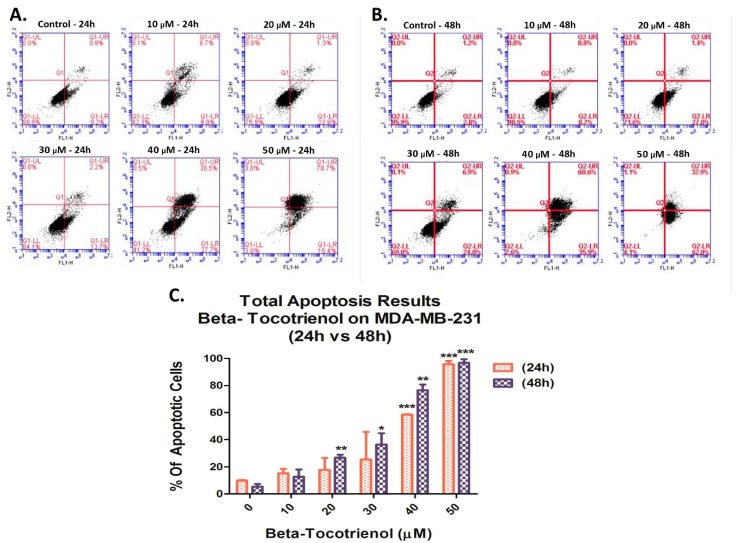
Beta-Tocotrienol induced apoptosis in MDA-MB-231 cells. A dual staining with Annexin V-FITC (FL1-H) and propidium iodide (FL2-H) was assessed to measure the amount of apoptosis of MDA-MB-231 cells by flow cytometry. Results were obtained after 24 and 48 h treatment periods, in (**A**) and (**B**) figures respectively by C-Flow software. The percentages of total apoptosis were quantified upon beta-T3 treatment and presented in histograms (**C**). *, ** and *** indicate *p* < 0.05, *p* < 0.001 and *p* < 0.0001 respectively.

**Figure 6:**
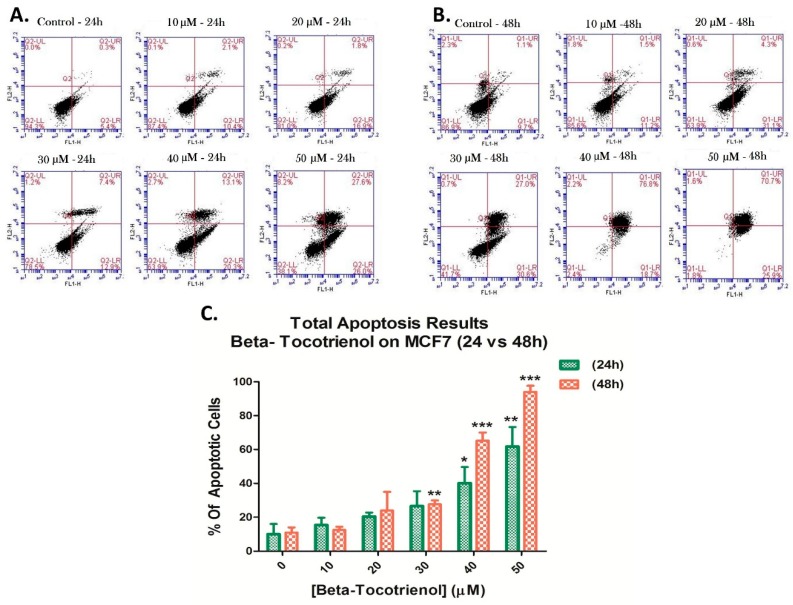
Beta-Tocotrienol induced apoptosis in MCF7 cells. A dual staining with Annexin V-FITC (FL1-H) and propidium iodide (FL2-H) was assessed to measure the amount of apoptosis of MCF7 cells by flow cytometry, upon 24 h (**A**) and 48 h (**B**) of treatment. Results were obtained by C-Flow software. The percentages of total apoptosis were quantified upon beta-T3 treatment and presented in histograms (**C**). *, ** and *** indicate *p* < 0.05, *p* < 0.001 and *p* < 0.0001 respectively.

Due to the observed decrease in cellular proliferation and the increase in sub-G1 population, dual Annexin V/PI staining was performed on both MDA-MB-231 and MCF7 cells treated with beta vitamin E isomer for 24 and 48 h, to examine the tocotrienol potential pro-apoptotic effects. Cells were then analyzed using flow cytometry. Figure 5 and Figure 6 revealed that beta-T3 triggered a dose- and time-dependent elevation in the apoptotic percentages of both BC cell lines.

When applied on MDA cells for 24 h, beta-T3 started to induce a significant increase in the percentage of apoptotic cells at 40 μM and 50 μM concentrations whereby early and late apoptosis measured 58.2% and 94.1% respectively, as compared to control untreated cells (10.1%), with a *p* < 0.0001 (Figure 5A,C). In addition, apoptosis percentages increased significantly between control and the 50 μM treated cells, respectively with 4% and 94.9 % of apoptosis following treatment for 48 h (Figure 5B,C). 

The flow cytometric analysis of MCF7 cells was similar to the results obtained in MDA cells. Viability percentages dropped significantly with increasing doses of beta-T3 starting at the 30 μM dose. However, the total apoptotic cell percentages of MCF7 were lower than those obtained for MDA; values varying from 5.7% in MCF7′s controls to 53.6% in 50 μM treated cells for 24 h (Figure 6A,C), and from 10.8% in controls to 96.6 % of apoptotic cells in 50 μM beta treated cells for 48 h (Figure 6B,C).

### 3.4. The Vitamin E Beta-T3 Triggers Pro-Apoptotic Proteins Up-Regulation by a p53- Independent Mechanism in the MDA-MB-231 Cell Line

Since higher anti-proliferative and pro-apoptotic effects were observed in MDA-MB-231 than in MCF7 cells, we then aimed to decipher the molecular mechanism underlying the effect of beta-T3 on apoptosis induction, and cell growth inhibition in MDA-MB-231 breast cancer cells. We tested the expression of several proteins involved in the apoptotic machinery (Bax, Bcl-2, p53, cytochrome C, cleaved PARP-1 and cleaved caspase-3) as well as the expression of two key cell proliferation proteins (p-PI3K and p-GSK-3), using β-actin as a loading control. Protein extraction from 24 h treated MDA-MB-231 cells was carried out using the concentrations 0, 10, 20 and 30 μM of beta-T3, since the IC50 value reported was 29.99 μM. 

**Figure 7:**
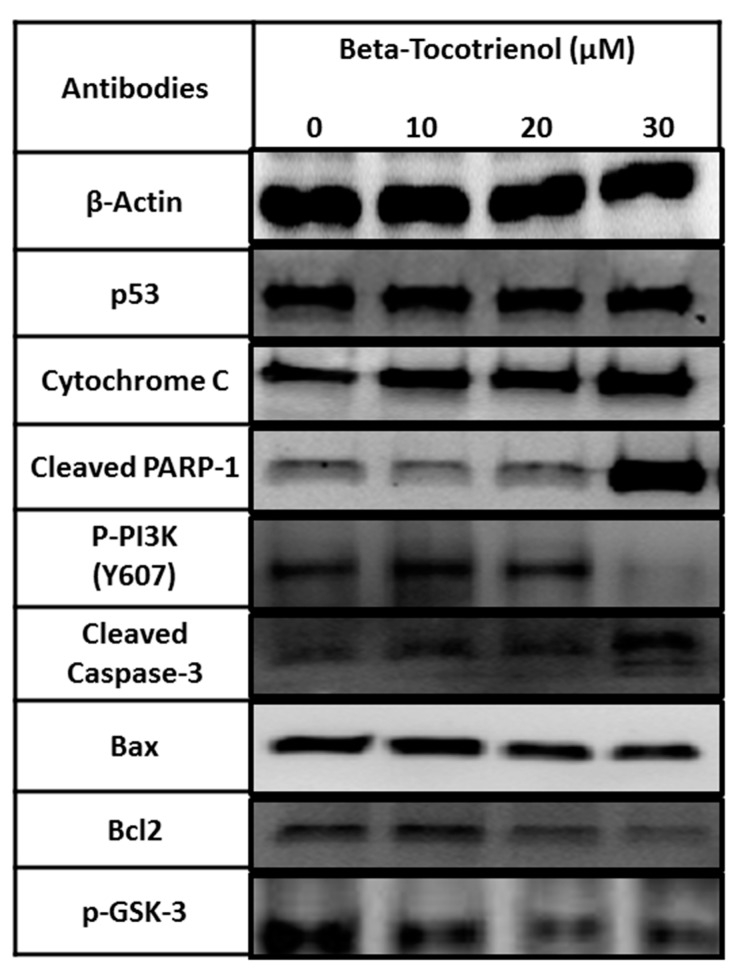
Beta-Tocotrienol remarkably up-regulates the pro-apoptotic protein expression of cytochrome c, increased caspase-3, and PARP-1 cleavages and downregulates Bcl-2 and two proliferation mediating proteins: p-PI3K and p-GSK3 in 24 h treated MDA-MB-231 cell lines. Western blot analysis of Bax, Bcl-2, p53, cytochrome C, cleaved PARP-1, cleaved caspase-3, p-PI3K, p-GSK3 and β-actin from untreated control and 10, 20 and 30 μM treated MDA-MB-231 breast cancer cells.

**Figure 8:**
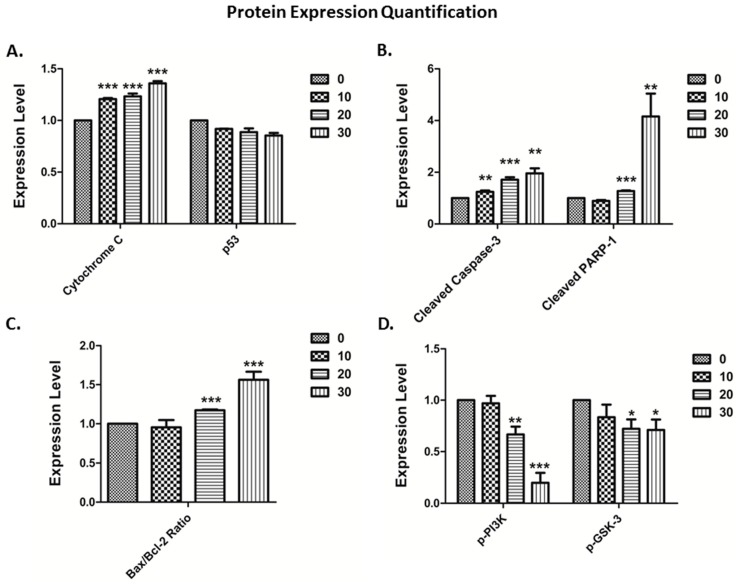
Quantification of apoptotic protein expression in MDA-MB-231 cells treated with beta-T3 vitamin E isomer for 24 h by the Image J software. Compared to control levels, beta-T3 significantly increased, on the one hand, cytochrome-c (**A**), cleaved caspase-3, cleaved-PARP-1 (**B**) and Bax/Bcl-2 ratio (**C**) expression levels. On the other hand, it significantly decreased the expression levels of two proliferation involved proteins p-PI3K and p-GSK-3 (**D**). *, ** and *** indicate *p* < 0.05, *p* < 0.001 and *p* < 0.0001 respectively.

Referring to Figure 7, beta-T3 was found to up-regulate significantly the expression of cytochrome C and increase the cleavage of caspase-3 and PARP-1proteins (Figure 8A,B). Moreover, it induced a downregulation in Bcl-2 expression without affecting neither Bax nor p53 expression levels. Bax/Bcl-2 ratio significantly increased between the untreated control and the cells treated with 20 and 30 μM beta-T3. Furthermore, beta-T3 treatment was able to induce a significant decrease in the expression of activated proliferation-related proteins p-PI3K and p-GSK-3 (Figure 8D). 

## 4. Discussion

Several studies on vitamin E derivatives revealed that tocotrienols have a superior biopotency over tocopherols especially with their higher cellular uptake enhanced by the double bonds present in the tocotrienol phytyl chain [53]. Recent data have pointed on the promising anti-cancer effects of tocotrienols including both anti-proliferative and apoptotic activity that can be effective against various types of cancer, such as liver [37,54], colon [55], lung [56], breast [57,58], skin [56], prostate [34,35], and even hematologic malignancies [55].

Although many studies in the literature have reported the anti-tumor effect of T3 in vitro and in vivo [29,59], very few studies have focused on the potency of beta-tocotrienol in this respect. Beta-T3 is known for its low abundance in nature, which makes its extraction difficult; however, it is found to be the predominant vitamin E form in all 6 tested varieties of whole wheat with amounts ranging from 0.9 to 1.9 mg /100 mg of wheat [9]. It is also the essential tocotrienol present in the black cumin oil with 1190 mg of beta-T3 / 100 g oil [60].

Until recently, Lim and his colleagues were the only ones who shed the light on the anti-cancer effect of beta-T3 against human lung and brain cancer cell lines [45]. Hence, in our present work, we aimed to investigate the anti-cancer effect of beta-T3 on two breast cancer cell lines, namely MDA-MB-231 and MCF7.

In the first part of our study, we investigated the anti-proliferative effects of beta-T3, and then compared them to those of the potent, widely studied isoform, gamma-T3 [12]. Results showed that both di-methylated ring vitamin E isoforms significantly decreased the growth of MDA-MB-231 and MCF7 cells in a dose- and time-dependent manner. Furthermore, both treatments exhibited a higher effect on MDA-MB-231 than MCF7 cells, IC 50 being equal to 21.14 μM and 24.34 μM upon beta-T3 treatment for 48 h and 30.98 and 32.87 μM upon gamma-T3 treatment for the same period of time on MDA-MB-231 and MCF7 respectively. This is in accordance with what was obtained by Loganathan et al. who reported higher IC50 values of gamma-T3 on MDA as compared to MCF7 cells [61]. This result is pivotal knowing that the treatment targets MDA-MB-231, a TNBC cell type known for its poor prognosis and high aggressiveness due to the absence of the estrogen hormone receptor. On the other hand, MCF7 and other (ER)-positive tumor types greatly benefit from hormonal therapies namely anti-estrogens [62,63]. The interesting findings in our study highlight the potent anti-cancer effect of beta-T3 on both BC cells, with more prominent effects observed upon beta-T3 than gamma-T3.

Moreover, aiming to investigate the mechanisms underlying the reduction in cellular viability, PI staining was used and showed that beta- T3 induced a mild G1 phase arrest in BC cells after 48 h in TNBC cell line MDA-MB-231 and at both 24 and 48 h for the ER-positive cell line, MCF7. This could be correlated with Patascil et al.’s result on the gamma-T3 isoform, the most similar in structure to beta-T3, which was proved to induce a G1 arrest in MCF7 cell lines via the down-regulation of cyclin D1/CDK4 kinase and the reduction of Rb phosphorylation [64]. Similarly to Lim et al., beta-T3 also triggered a sub-G1 upsurge in cell population, especially at 40 and 50 μM concentrations, indicating apoptosis induction [45]. 

Next, we proceeded to certify the apoptotic route of cell death using Annexin/PI double staining, which revealed a drastic increase in the percentages of apoptotic cells in both BC cell lines, after both 24 and 48 h of treatment. Accordingly, the observed effect of beta-T3 was similar to that observed in other tocotrienol vitamin E members in inducing apoptotic breast cancer cell death, in vitro, regardless of the presence of estrogen receptors [57,64,65].

Furthermore, in line with or findings, Lim et al. have previously reported the pro-apoptotic effect of beta-T3 marked by morphological features such as chromatin condensation, cell shrinkage, cytoplasm vacuolization, and nuclear membrane permeability loss, on both A549 and U87MG cells, compared to the untreated controls without inducing any necrotic effects [45].

Being more responsive than MCF7 upon treatment with beta-T3, MDA-MB-231 cells were then chosen to investigate the underlying molecular mechanism of action using westerns blots to target some proteins involved in apoptosis and cell proliferation. In fact, previous reports suggested that multiple pathways can be regulated by tocotrienols, acting against various types of cancer: anti-inflammatory, anti-proliferative, pro-apoptotic, anti-metastatic and anti-angiogenic pathways [66]. Apoptosis signaling englobes two main pathways, the first one is the extrinsic pathway, in which cell death ligands bind their surface-receptors, and recruit specific proteases called caspases, that can be activated by cleavage, starting with the initiator caspase 8. The second one is the intrinsic pathway triggered via cellular stresses, mainly the mitochondrial stress controlled by several proteins such as the Bcl-2 family, namely Bax and Bcl-2, leading to cytochrome c release into the cytoplasm, then the activation of caspase-9 as an initiator protease.

Both pathways merge together in such a way that, once the initiator caspases are activated, they recruit subsequent cleavage of effector caspases (starting with caspase-3) that are, in turn, able to cleave various protein substrates leading to apoptotic cell death [57,67]. The Poly (ADP-ribose) Polymerase (PARP) constitutes one of these substrates that after cleavage, induces the ribosylation of numerous nuclear proteins involved in the apoptotic pathway [67,68]. Within the past years, intensive research on tocotrienols, especially against breast cancer, has indicated that these isoprenoids can inhibit the tumor necrosis factor-alpha (TNF- α), activate p53, deactivate NF- kB which enable the downregulation of many gene products related to cell survival such as apoptosis inhibitors B-cell lym-phoma 2 (Bcl-2), proteins (IAP)-1, −2, B-cell lymphoma-extra-large (Bcl-xL), X-linked inhibitor of apoptosis (XIAP), survivin, cellular FLICE-like inhibitory protein (cFLIP), etc [59,69]. Other studies have also shown that cell death can involve caspase-8-mediated extrinsic apoptosis, ER or mitochondrial stress -intrinsic apoptotic pathway, and autophagy [45,64,70,71,72,73,74].

The analysis of our blots revealed that the apoptotic effect of beta-tocotrienol on MDA-MB-231 cells involves the up-regulation of cytochrome c, the cleavage of caspase-3 and PARP-1 protein, the down-regulation of the anti-apoptotic protein Bcl-2, without affecting the expression of the apoptotic protein Bax. However, the bax/bcl-2 ratio was significantly increased. Additionally, the beta-T3 apoptotic effect did not require any involvement of the p53 pathway, the p53 expression being non-modified. These results are consistent with previously reported data on gamma-T3 found to inhibit cell proliferation in human breast cancer cells by inducing the expression of Bcl-2 family proteins, and increasing cytochrome c release, caspases-9, and -3 activation and PARP fragmentation [38,64,68].

Initial studies on tocotrienols showed an important association between the decrease in the Pl3K/Akt pathway and the apoptosis trigger in mouse mammary cells [53,75]. In fact, PI3K /AKT signaling constitute a potential therapeutic target involved in growth, survival and invasion mediation in various types of cancers including breast carcinomas [76,77]. In correlation with Sylvester’s finding on gamma-T3 [7], our data revealed that treating BC cells with beta-T3 triggered a downregulation in the expression of the Y607- phosphorylated PI3K protein.

Moreover, we tested the effect of the beta-T3 on the expression of GSK-3 (or Glycogen Synthase Kinase-3), a key enzyme for glycogen metabolism, and a serine/threonine protein kinase that regulates many cellular functions [78,79]. In human breast cancer, overexpression of GSK-3β is associated with poor prognosis indicators (increased tumor size, ER-negative disease, high pathological grade, PR-negative disease, and relapse after tumor resection) [80]. Similarly to gamma-T3′s effect in reducing phosphorylated GSK3α/β [81,82,83,84], our beta-T3 isoform showed a decrease in the auto-phosphorylation of Tyr279 in GSK3α and Tyr216 in GSK3β expression levels leading to a decrease in GSK-3 activity [85,86].

To brief up using a head-head comparison, Patascil et al. [64] showed, in consistence with our used range of concentrations, that gamma-T3 was able to inhibit the cell proliferation in a dose- and time--dependent manner of both breast cancer cell lines involved in our study, then they demonstrate that a 40 μM of gamma-T3 was able to induce a modest G1-phase arrest associated with cyclin D1/D3 and CDK4 down-regulation, upon 24 h treatment in the MCF7 cells. In parallel, Beta-T3 was able to induce a G1 arrest at lower concentrations of 20 and 30 μM after 24 h on the same cell line. Further investigations are needed to discover the potential implied cell cycle players in this ER(+ve) BC cell line. Also, Gamma-T3 was found to trigger ER-stress apoptotic cell death via modulating many involved genes and proteins expression such as activating transcription factor 3, ATF3, protein kinase-like endoplasmic reticulum kinase PERK and inositol requiring kinase 1 α, in both MCF-7 and MDA-MB 231cells. In addition, gamma-T3 treatment increased cleaved – PARP and caspase-7 protein levels [64]. As well, ParK and colleagues reported that γ-T3 induced cleavage of PARP, and caspases-8, -9, and -3, revealing that gamma-T3 can mediate several apoptotic routes [72], without modifying the Bax/Bcl-2 ratio in treated MDA-MB-231 neither at 24 h nor at 48 h [38]. Moving to the beta-T3 vitamin E member, it was found to trigger extrinsic apoptotic cell death via caspase-8-mediated Bid and Bax activations [45], and intrinsic p53-independent apoptotic pathway as revealed in our study particularly at concentrations starting from 20 μM of beta-T3, the Bax/Bcl-2 ratio being significantly increased; however, the ER-stress-mediated apoptosis induction by beta-T3 remains unproven to date.

In conclusion, the data presented reveal the potent pro-apoptotic effect of beta-T3 in BC cell lines in vitro, highlighting the need for further studies to confirm its effects in vitro. One limitation of our study is the unavailability of normal cell lines to test the specificity of beta-T3; promisingly, an earlier study has reported no effects of a tocotrienol rich fraction from palm oil, which is rich in beta-T3, on normal cell lines [34]. It is noteworthy that subsequent in vivo and clinical studies using beta-T3 will require specific delivery systems as previously suggested for tocotrienols. As reported by Maniam et al., the use of tocotrienols is hampered by their lower bioavailability, solubility, and poor pharmacokinetic profile when compared to tocopherols [87,88]. Despite the fact that oral T3s have better bioavailability in rats than intramuscular and intra-peritoneal routes of administration, this bioavailability couldn’t exceed more than 28% in the case of alpha-T3, and not more than 9% for γ- and δ-tocotrienols [59,89]. In order to overcome these challenges, nanotechnology delivery tools were developed to improve tocotrienols efficacy in vivo. Using nano-formulations (tumor-targeted nano-carriers) in animal models, tocotrienols exhibited a 10-fold higher anti-proliferative effect and resulted in up to 60% of tumor regression [87]. Thus, improving tocotrienols, especially beta-T3′s, bio-absorption and access to tumor cells through nanotechnology bio-systems, will provide an enhanced therapeutic effect than usual delivery routes.

## 5. Conclusions

In conclusion, the beta-tocotrienol vitamin E member was found to exhibit a promising anti-tumorigenic effect more pronounced than gamma-tocotrienol on breast cancer models in vitro. It triggers a mild G1 phase cell cycle arrest, and its apoptotic effect is induced through a p53-independent apoptotic mechanism. Further studies are needed to investigate the effect of beta-T3 on other cancer types in vitro and in vivo, which may widen the horizon for promising combinations with chemotherapeutic drugs.

## Figures and Tables

**Table 1:** Summary of IC50 values upon treatment of breast cancer cells MDA-MB-231 and MCF7 with a range of concentration (0-50 μM) of beta- or gamma-tocotrienols for 24 and 48 h.

	Beta-Tocotrienol IC50 (μM)		Gamma-Tocotrienol IC50 (μM)	
	24 h	48 h	24 h	48 h
**MDA-MB-231**	30.0 ± 1.8	21.1 ± 2.8	39.0 ± 5.5 *	31.0 ± 3.9 **
**MCF7**	30.5 ± 2.3	24.3 ± 1.3	41.1 ± 3.9 **	32.9 ± 3.6 **

Values are presented as averages ± SD. * and ** indicate for each cell line significance between mean values of IC50 of the two different treatments and after the same time duration, respectively if *p* < 0.05 and *p* < 0.001.
